# Did relaxing clinical trial regulation enhance the stock of scientific knowledge in India? Not necessarily

**DOI:** 10.1371/journal.pone.0210163

**Published:** 2019-01-03

**Authors:** Bastian Rake, Carolin Haeussler

**Affiliations:** 1 School of Business, Maynooth University, Maynooth, Co Kildare, Ireland; 2 Chair of Organisation, Technology Management and Entrepreneurship, University of Passau, Passau, Germany; York University, CANADA

## Abstract

The increasing amount of clinical research conducted outside the “traditional” countries raises questions about the benefits of hosting offshored clinical research. The extent to which trials contribute to the scientific knowledge base and, in particular, whether there are differences between different types of trials remain open questions. By examining a change in clinical trial regulations in India, a country often viewed as a first-choice offshoring location, we study how the relaxation of clinical trial regulations affects the number and the type of clinical trials as well as the domestic scientific knowledge base. Based on trial data from ClinicalTrials.gov and data on associated publication activities, our empirical analysis suggests that, despite an initial increase in the number of clinical trials, relaxing clinical trial regulations has a limited impact on the domestic scientific knowledge base. More specifically, the number of Indian researchers involved in the production of trial-related scientific knowledge remains modest. Furthermore, the potential to learn from the additional trials appears to be limited: the influx of phase 3 trials—mainly sponsored by Western-pharmaceutical firms—is accompanied by a lower likelihood that the trial results will be used in Indian researchers’ subsequent research activities when compared to phase 3 trials with preceding phase 2 trials, as was required before the regulatory change. Overall, our results contradict expectations that relaxing the regulatory requirements for conducting late-stage clinical trials is an appropriate means of supporting the development of the domestic scientific knowledge base.

## Introduction

The geographical distribution of clinical research has changed considerably over time, such that clinical trials are increasingly conducted outside the “traditional” research centers in North America, Western Europe, and selected locations in the Asia-Pacific region. Non-traditional countries, particularly emerging economies in East Asia, Southeast Asia, and Latin America, have gained importance as locations for conducting clinical trials [1–4]. This rising importance of non-traditional countries coincides with an increase in intercontinental industry-sponsored trials [5]. Given pharmaceutical firms’ interest in outsourcing clinical trials to emerging economies, national governments face the complex decision of whether to hamper or support this development through, for example, policy decisions, public investments in research infrastructure, or regulatory changes [6,7]. Arguments for restricting (offshored) clinical trials include the crowding out of domestic research, and concerns about patient safety, access to information about the research, and the possibility of a lack of access to medications after a trial has been completed when those medications have been tested on the country’s population [2,8,9]. In addition, researchers have suggested that offshored clinical trials often focus on diseases that are predominantly prevalent in high-income countries, while they largely ignore diseases common in low-income countries [10,11]. In contrast, arguments for attracting clinical trials include the enhanced availability of new medications that correspond to local needs, the support of domestic health systems, and opportunities for knowledge transfer and learning [9,2].

Although knowledge-transfer arguments are highlighted in policy discussions and reports, the extent to which countries’ scientific knowledge bases truly benefit from attracting clinical research sponsored by Western organizations is still an open question. Anecdotal evidence suggests that an influx of trials strengthens the host country’s scientific capabilities, but there is a lack of corresponding quantitative analyses. To address this gap, we study how relaxing clinical trial regulations affects the number of clinical trials as well as the domestic scientific knowledge base in a country by measuring scientific publications and forward citations. More specifically, we analyze whether a policy change aimed at increasing late-stage clinical trials in an emerging economy fosters the involvement of domestic researchers in the production of scientific knowledge. In particular, we investigate the change in trial-related publication output as well as the dissemination of trial-related knowledge based on forward citations by domestic authors. Since a thorough evaluation of the policy change would require including a large number of aspects ranging from patient safety to market aspects and is beyond the scope of a single paper, we focus on how the production of scientific knowledge in terms of publications and their use by domestic researchers through forward citations may be affected.

Our study’s context is a country that has long been a first-choice offshoring location [12–15]: India. To increase its attractiveness for (offshored) clinical trials, India repealed its so-called “phase lag” regulation, which applied to compounds not discovered in India, in January 2005 [16,8]. According to the pre-2005 regulation, clinical trials in India had to be conducted in an earlier drug-development phase than the phase of trials being undertaken in other countries for the same drug. If, for example, a new drug was being tested in a phase 3 study in the United States, the same drug could only be tested in a phase 2 study in India. Table 1 provides an overview of the key change in India’s regulatory framework in 2005. In order to assess the consequences of this regulatory change, it is important to take a closer look at the context in which it took place.

**Table 1:** Overview of key change in India’s clinical trial regulations.

Clinical Trial Regulations Prior to January 2005	Clinical Trial Regulations Since January 2005
Phase 1 trials of compounds not discovered in India were generally not allowed. Trials in later phases for compounds not discovered in India needed to be initiated with a “phase lag.”	Phase 1 trials of compounds not discovered in India are generally not allowed. Trials in later phases for compounds not discovered in India can be initiated at the same time as in other countries.

After its independence in 1947, India adopted a self-reliance policy based on price controls and other regulations aimed at supporting its domestic pharmaceutical industry. The policy included high tariffs and foreign ownership restrictions, which limited dependence on Western pharmaceutical companies with respect to drug development and the supply of pharmaceuticals [17]. Furthermore, starting in the early 1970s, product patents for pharmaceuticals could no longer be filed, and patents for pharmaceutical production processes expired seven years from the application date or five years after they were granted [18,19]. In addition, India’s regulatory framework for clinical trials was designed to protect the domestic population from being abused as experimental subjects for untested and possibly unsafe drugs developed by foreign companies, especially those of Western origin [8]. Consequently, phase 1 trials of compounds not discovered in India were generally not allowed and trials in other stages needed to be initiated after a “phase lag.”

This combination of regulations enabled Indian companies to improve their position in both the domestic market and overseas, primarily based on the development of generic varieties of medications originally developed and patented by Western companies [20]. However, this imitation-based business model came into question in the 1990s when India became a member of the World Trade Organization and was required to comply with the TRIPS agreement. As a consequence, the country had to introduce product patents by 2005 [21–23]. Consequently, the industry had to adapt its business model. Alternatives in this regard included imitating off-patent drugs or developing new drugs based on India’s own R&D activities. Furthermore, the Indian pharmaceutical industry was challenged to build up the biotechnology skills needed to develop innovative drugs [24,25].

The Indian Pharmaceutical Research & Development Committee [26] arrived at the conclusion that changes in clinical trial regulations could turn India into a worldwide leading location for clinical trials. This potential for clinical research was expected to support the transformation of the domestic pharmaceutical industry from a”late follower to an innovative leader” by encouraging the return of Indian scientists from abroad, the establishment of knowledge-intensive R&D service companies, and to increase foreign direct investment (FDI) by multi-national pharmaceutical companies. In January 2005, the Indian government finally amended its Drug and Cosmetic Rules and repealed the”phase lag” for compounds not discovered in India [16,8]. Indian companies reacted favorably to these changes based on the expectation that the changes would strengthen the country’s scientific and innovation capacity, and increase profits by enabling Indian companies to provide services to the Western pharmaceutical firms expected to offshore trials to India [27]. In contrast to the positive expectations of the Indian government and domestic companies, the national media stressed the potential risks for Indian trial subjects, who might be abused as “guinea pigs” by multinational companies [28,29].

Against this background, Reid and Ramani [30] point out that conducting clinical research has been viewed as key for India’s ability to catch up to the international knowledge frontier. However, whether the changes resulted in the expected catch-up effect is unknown. It may well be that clinical research has not enabled India to develop the scientific capabilities that are required for pre-clinical or upstream research. As such, activities linked to drug discovery may still only be conducted in R&D labs outside of India.

In this paper, we provide a thorough analysis of the consequences of the regulatory changes for India’s domestic scientific knowledge base. To do so, we investigate the relation between trials of new medications that correspond to international demands and changes in India’s scientific knowledge base. In line with prior studies, we build our analysis on measurements of scientific publications and forward citations to account for the scientific knowledge base [31]. Scientific publications document scientific discoveries and the creation of new knowledge that advances the existing knowledge base in a specific area of research. Forward citations reflect the use of a published piece of knowledge by other researchers in their own attempts to contribute to the scientific knowledge base [32]. Against this background, we investigate the extent to which the repeal of the phase lag strengthened the involvement of Indian researchers in publications related to clinical trials and the extent to which Indian researchers have been able to build on trial-related publications.

## Methods

### Clinical trials and publication data

Our empirical analysis, which explores the consequences of the repeal of the “phase lag” for India’s domestic scientific knowledge base, is based on clinical trial data obtained from ClinicalTrials.gov. That dataset contains detailed information about clinical trials conducted in the US and 179 other countries, including India and many other non-traditional countries. Haeussler and Rake [1] provide a detailed overview of the database’s characteristics, the types of clinical trials registered in ClinicalTrials.gov, and the regulatory requirements for registration. As we focus on India, our dataset encompasses 725 phase 3 clinical trials. Phase 3 trials use comparatively large samples of trial subjects to evaluate the safety and efficacy of various doses of drugs or biological products for different populations. The trials covered in our dataset were launched between January 2002 and December 2012, and were conducted in at least one facility located in India, usually hospitals or other medical-care institutions. Of the 725 phase 3 trials, 722 started after the repeal of the phase lag requirement (i.e., between January 2005 and December 2012).

We identified whether a phase 3 trial was preceded by a phase 2 trial in India. Phase 2 trials test drug candidates in humans who are affected by specific diseases or conditions. The objective is to obtain preliminary data on effectiveness and adverse events that occur in the short term. As ClinicalTrials.gov does not provide unique identifiers for each drug under development, we relied on the work of six experts in biology and medicine to create drug synonym groups. Drug synonym groups uniquely identify drugs that are under development, as they are labeled differently during the different phases of their development. Put differently, drug synonym groups enable us to identify whether the same drug had been tested in different trials using different names or labels. By matching each clinical trial with the corresponding drug synonym group, we identified whether a phase 3 trial was preceded by a phase 2 trial of the same compound in India.

ClinicalTrials.gov provides information about the type of (lead) sponsor of each clinical trial. This information allowed us to distinguish clinical trials that are sponsored by biotechnology and pharmaceutical companies from those that are sponsored by academic institutions or other organizations.

**Fig 1:**
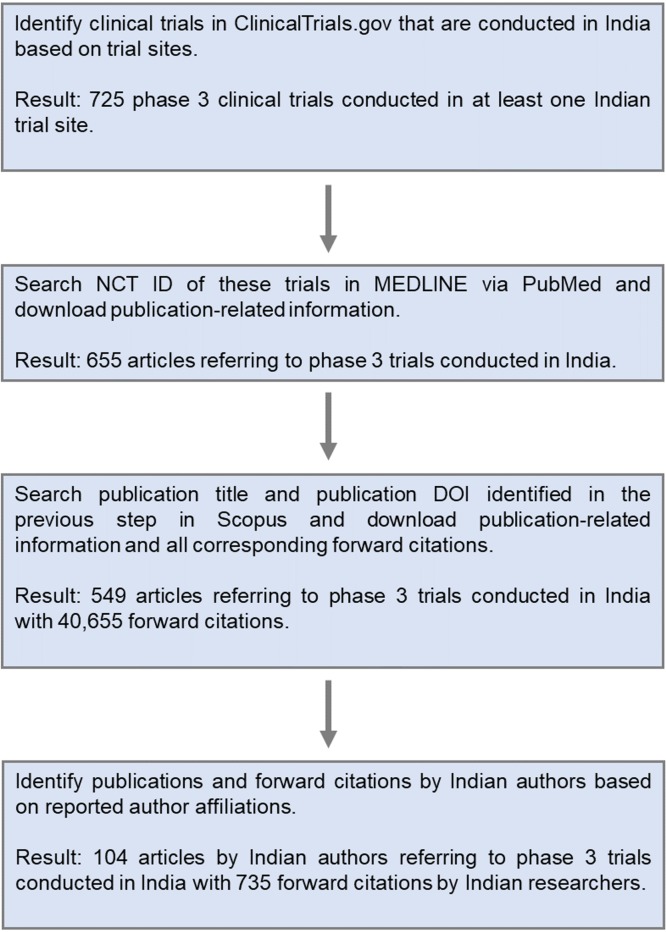
Search strategy for publication data.

In order to account for the involvement of researchers with Indian affiliations in publications related to clinical trials, we followed Hoekman et al. [33] and searched the trial registration numbers in MEDLINE via PubMed (see Fig 1). MEDLINE indexes more than 5,500 journals, including the leading life-science and biomedical scientific journals as well as domestic scientific journals from India and other countries. As MEDLINE does not provide information on all author affiliations, we complemented the data with information on author affiliations from Scopus, one of the world’s largest abstract and citation databases covering peer-reviewed research. We found 549 articles that list a trial-registration number referring to a phase 3 trial conducted in India. Of these, 104 had at least one author with an Indian affiliation. These articles either report the intermediate or final results of a particular clinical trial, or describe trial protocols and the research methods used in a clinical trial. Scopus can also be used to obtain detailed information concerning scientific articles that cite trial-related publications. In this regard, we found 40,655 citations referring to the trial-related publications in our sample. 735 of these 40,655 citations had at least one author affiliated with an Indian institution.

In order to obtain information on the type of trial-related publications and the citing publications related to the clinical trials in our sample, we use the CHI journal-classification system [34]. The CHI system assigns scientific journals to one of four categories depending on whether the articles in those journals can be described as predominantly focused on basic research or clinical research. Hence, the CHI journal-classification system enables us to obtain an indicator of whether a trial-related publication has been published in a journal that is predominantly focused on basic or clinical research; this may impact how often a publication is cited.

## Analyses

We use descriptive analyses to investigate developments in the number of phase 3 trials conducted in India with and without preceding phase 2 trials as well as the number of publications related to those trials and their forward citations. In addition, we use regression analysis to examine the consequences of the repeal of the phase lag requirement on the Indian scientific knowledge base. Our regression analysis is based on a sample of 385 publications related to phase 3 trials conducted in India that started between the enactment of the regulatory changes in January 2005 and December 2012. Note that we lost observations because publications could not be matched to the CHI classification system or there was no information about the length of the publication available. As a dependent variable, we used how often a trial-related publication was cited in subsequent publications that listed at least one author with an Indian affiliation. This measure allows us to examine the impact of trial-related publications within the Indian science system, as shown in Models 1, 2, and 3. In Models 4, 5, and 6, we included only those forward citations associated with at least one author from India that were made in the first three years following the publication of the focal phase 3 trial-related publication. The dependent variable is a count variable, i.e., a variable which can take only non-negative integer values including zero. Therefore, we use negative binomial regressions with Huber-White robust standard errors, which is a standard model for analyzing count data. All regressions include several publication- and trial-related control variables. An overview of the variables can be found in Table 2.

**Table 2:** Description of variables and summary statistics.

Variable	Description	N	Mean	SD	Min.	Max.
Citations by Indian authors	Number of forward citations reporting at least one author affiliation in India	385	1.60	3.90	0	41
Citations by Indian authors in the first three years after publication	Number of forward citations reporting at least one author affiliation in India in the first three years after publication of the focal publication	385	1.21	2.63	0	26
Weighted citations by Indian authors	Weighted number of forward citations reporting at least one author affiliation in India	385	1.05	2.77	0	29.62
Weighted citations by Indian authors in the first three years after publication	Weighted number of forward citations reporting at least one author affiliation in India in the first three years after publication of the focal publication	385	0.78	1.89	0	19
Indian author	Dummy variable indicating the presence or lack of at least one author affiliation in India	385	0.18	0.38	0	1
No preceding phase 2	Dummy variable indicating whether the phase 3 trial had no preceding phase 2 trial conducted in India	385	0.86	0.35	0	1
Basic research journal	Dummy variable indicating whether the publication appeared in a journal classified as “basic biomedical research” or “clinical investigation” according to the CHI journal classification	385	0.11	0.31	0	1
Number of authors	Number of authors of the article	385	12.95	20.77	1	373
Page count	Number of pages in the article	385	8.20	2.36	1	23
Number of countries (publication)	Number of countries involved in the publication according to author affiliations	385	5.26	4.44	1	40
Traditional country co-author	Dummy variable indicating whether at least one author affiliation is in a traditional clinical trial country	385	0.96	0.20	0	1
Life-threatening disease	Clinical trial addresses a life-threatening disease according to FDA regulations	385	0.50	0.50	0	1
Industry sponsor	Clinical trial sponsored by a company	385	0.91	0.29	0	1
Number of countries (trial)	Number of countries in which the clinical trial is conducted	385	20.23	10.74	1	46
Domestic sponsor	Clinical trial sponsored by an organization based in India	385	0.05	0.22	0	1

In Models 7, 8, and 9 we use a weighted count of forward citations found in publications with at least one author affiliated with India as the dependent variable. More specifically, we weighted each publication by the number of countries listed in the authors’ affiliations in order to account for the contribution of Indian authors to each forward citation. Consequently, a forward citation that lists India and two other countries in the author affiliations would contribute 1/3 to India’s weighted citation count. In Models 10, 11, and 12, we include only weighted forward citations that were made in the first three years after publication of the focal article. We analyzed the models using weighted citations as the dependent variable, and we relied on Tobit regression models with Huber-White robust standard errors. These models are suitable when data entries for a considerable number of cases are equal to zero and when the data are roughly continuously distributed over positive values. We use ordinary least squares regressions as an alternative method to assess the robustness of our findings for weighted citation counts. In addition, we use an auxiliary analysis to ensure that our results are not driven by clinical trials testing generic drugs or biosimilars. To carry out this analysis, we add a control variable to our analysis that indicates whether a clinical trial is sponsored by companies that exclusively produce generics and biosimilars or indicates the evaluation of generics or biosimilars in its official title or description.

Our aim is to investigate whether Indian authors cite publications related to phase 3 trials with or without preceding phase 2 trials more often.

## Results

### Clinical trials in India

**Fig 2:**
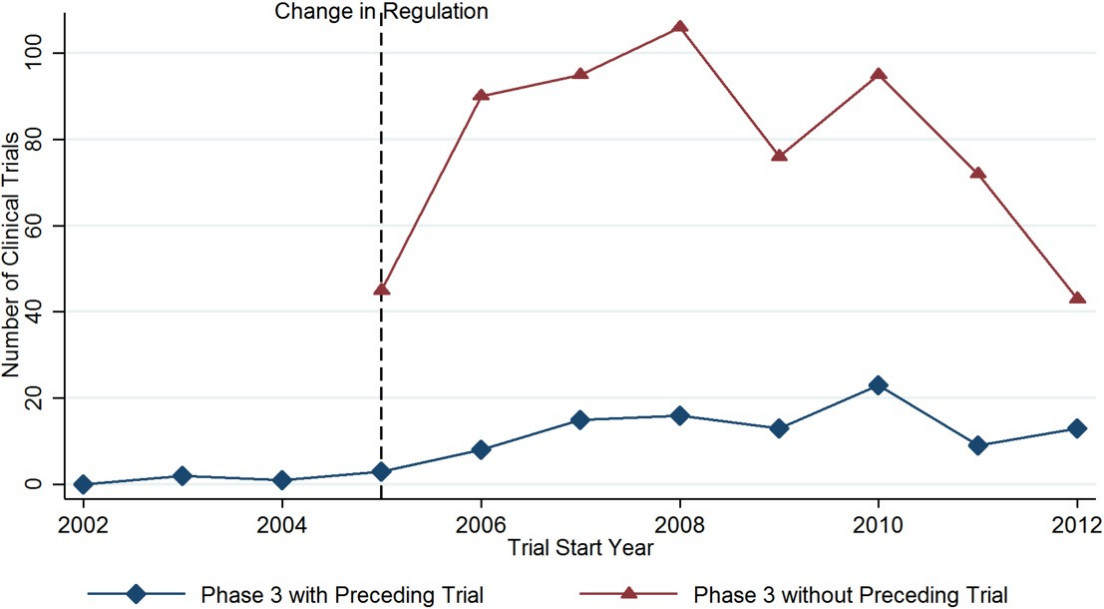
Phase 3 trials conducted in India.

With respect to the changes in phase 3 clinical trials over time, visual inspection of Fig 2 suggests that the repeal of the phase lag requirement enabled Indian researchers to conduct phase 3 trials without having to carry out a preceding phase 2 trials from 2005 on. In subsequent years, the number of phase 3 trials without preceding phase 2 trials increased considerably until 2008.

**Fig 3:**
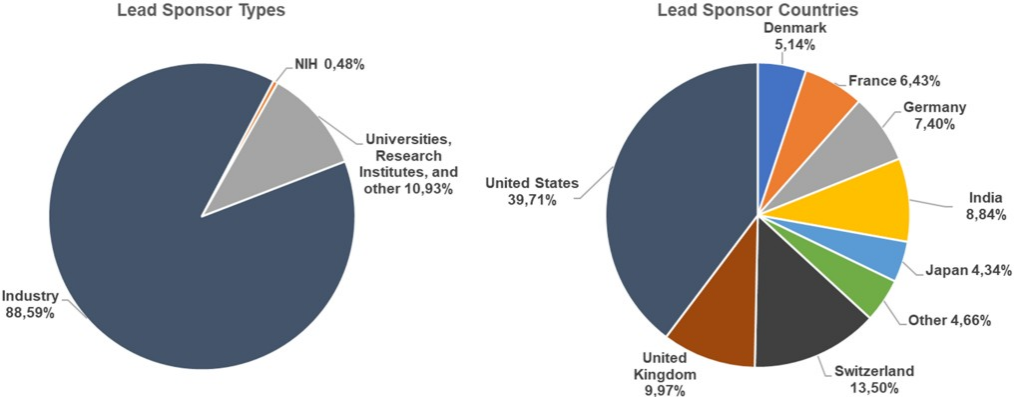
Sponsors of phase 3 trials conducted in India without preceding phase 2 trials.

The vast majority of phase 3 clinical trials without preceding phase 2 trials that started after 2005 were sponsored by biotechnology and pharmaceutical companies, while a minority were sponsored by universities and research institutes (see Fig 3). Importantly, most phase 3 trials were sponsored by companies and other organizations headquartered in traditional clinical research countries.

#### Relaxing trial regulation and the Indian scientific knowledge base—Descriptive results

We find that the absolute number of trial-related publications and the absolute number of citations of publications related to those trials increased over time (see Table 3). However, the involvement of Indian-based researchers in trial-related publication activities remained modest following the repeal of the phase lag. In order to account for the countries involved, we computed weighted publication and citations counts in which each publication or citation was weighted by the number of countries involved. The average number of weighted trial-related publications by Indian authors is slightly (but not significantly) higher for phase 3 trials that were not preceded by a phase 2 trial in India (0.053) (i.e., trials that were affected by the regulatory change) than for phase 3 trials that were preceded by a phase 2 trial (0.045). In contrast, the z-test indicates that, on average, India-based researchers refer in their follow-on work significantly more to phase 3 trial-related publications with preceding phase 2 trials conducted in India than to phase 3 trial-related publications without preceding phase 2 trials.

**Table 3:** Number of phase 3 trials, publications, and citations.

	Phase 3 trials with preceding phase 2 trials	Phase 3 trials with preceding phase 2 trials	Phase 3 Trials without preceding phase 2 trials	Industry-sponsored phase 3 trials with preceding phase 2 trials	Industry-sponsored phase 3 trials without preceding phase 2 trials
Trial start years	2002–2004	2005–2012	2005–2012	2005–2012	2005–2012
Number of trials	3	100	622	91	551
Number of publications	7	71	471	67	423
Number of publications by Indian authors	1	14	89	11	49
Number of weighted publications by Indian authors	0.143	4.474	33.252	2.640	12.012
Average number of publications per trial (Number of trial-related publications / Number of trials)	2.333	0.710	0.757	0.736	0.768
Average number of publications by Indian authors per trial (Number of trial-related publications by Indian authors / Number of trials)	0.333	0.140	0.143	0.121	0.089
Average number of weighted publications by Indian authors per trial (Number of weighted trial-related publications by Indian authors / Number of trials)	0.048	0.045	0.053	0.029	0.022
Number of citations	223	5,968	34,464	5,680	33,210
Number of citations by Indian authors	3	140	592	67	443
Number of weighted citations by Indian authors	0.411	97.565	366.820	42.449	281.478
Average number of citations per trial (Number of citations to trial-related publications / Number of trials)	74.333	59.680	55.408	62.418	60.272
Average number of citations by Indian authors per trial (Number of citations to trial-related publications by Indian authors / Number of trials)	1.000	1.400	0.952	0.736	0.804
Average number of weighted citations by Indian authors per trial (Number of weighted citations to trial-related publications by Indian authors / Number of trials)	0.137	0.976	0.590	0.466	0.511

Table 3 indicates that the engagement of Indian researchers in trial-related publications is generally lower for industry-sponsored trials (i.e., trials predominantly sponsored by Western multinational companies). In contrast to our findings for the entire sample, we find that for industry-sponsored trials, the average number of (weighted) publications by Indian authors for phase 3 trials without preceding phase 2 trials is lower than for trials with preceding phase 2 trials.

### Relaxing trial regulation and the Indian scientific knowledge base—Multivariate analysis

Our regression results, which are presented in Table 4, suggest that the number of citations by Indian authors increases when an author working for an Indian organization was involved in the publication related to a phase 3 clinical trial conducted in India. In the negative binomial regressions, we do not find significant associations between the absence of a preceding phase 2 study conducted in India and the number of citations by Indian researchers. However, we find a significant negative interaction between *Indian Author* and *No Preceding Phase 2* in Model 3, indicating that Indian authors cite trial-related publications less frequently when the corresponding phase 3 clinical trial did not have a phase 2 study conducted in India. However, this negative interaction disappears when we restrict the number of citations to forward citations made in the first three years after the publication of a trial-related study.

**Table 4:** Regression analysis of citations of trial-related publications by Indian authors.

	(1)	(2)	(3)	(4)	(5)	(6)	(7)	(8)	(9)	(10)	(11)	(12)
Regression model:	Negative binomial						Tobit					
Dependent variable:	Citations to trial-related publications by Indian authors			Citations by Indian authors in the first three years after publication			Weighted citations by Indian authors			Weighted citations by Indian authors in the first three years after publication		
Indian author	0.63***	0.62***	1.19***	0.59**	0.58**	1.12***	1.86**	1.88**	6.52***	1.24*	1.25*	4.85***
	(0.23)	(0.23)	(0.37)	(0.24)	(0.23)	(0.38)	(0.92)	(0.91)	(2.14)	(0.68)	(0.66)	(1.72)
No preceding phase 2		-0.17	0.09		-0.22	0.03		-1.78*	-0.17		-1.38*	-0.14
		(0.21)	(0.27)		(0.22)	(0.27)		(0.93)	(0.65)		(0.72)	(0.53)
Indian author*No preceding phase 2			-0.73*			-0.71			-5.83**			-4.55**
			(0.42)			(0.43)			(2.35)			(1.80)
Basic research journal	-0.17	-0.15	-0.09	-0.29	-0.27	-0.20	-0.86	-0.64	-0.34	-0.92	-0.74	-0.48
	(0.27)	(0.27)	(0.27)	(0.32)	(0.32)	(0.32)	(0.91)	(0.88)	(0.84)	(0.77)	(0.74)	(0.69)
Number of authors	-0.01	-0.01	-0.01	-0.01	-0.01	-0.01	-0.06	-0.06	-0.03	-0.02	-0.02	-0.01
	(0.00)	(0.00)	(0.00)	(0.00)	(0.00)	(0.00)	(0.06)	(0.06)	(0.04)	(0.03)	(0.03)	(0.01)
Page count	0.09**	0.09**	0.09**	0.09**	0.09**	0.09**	0.06	0.08	0.07	0.04	0.05	0.05
	(0.04)	(0.04)	(0.04)	(0.04)	(0.04)	(0.04)	(0.09)	(0.09)	(0.08)	(0.08)	(0.07)	(0.07)
Number of countries (publication)	0.05**	0.05**	0.06***	0.05**	0.05**	0.06**	0.16	0.13	0.12	0.10	0.08	0.08
	(0.02)	(0.02)	(0.02)	(0.02)	(0.02)	(0.02)	(0.11)	(0.11)	(0.09)	(0.07)	(0.07)	(0.05)
Traditional country co-author	1.18**	1.19**	1.08*	1.60***	1.60***	1.49***	6.90**	6.88**	6.22**	4.74**	4.77**	4.21***
	(0.56)	(0.57)	(0.57)	(0.52)	(0.53)	(0.55)	(3.38)	(3.05)	(2.62)	(2.08)	(1.87)	(1.58)
Life-threatening disease	0.42***	0.41***	0.42***	0.40**	0.38**	0.39**	0.85*	0.73	0.74	0.68*	0.58	0.59*
	(0.15)	(0.15)	(0.15)	(0.16)	(0.16)	(0.16)	(0.46)	(0.47)	(0.46)	(0.36)	(0.36)	(0.35)
Industry sponsor	-1.13***	-1.13***	-1.10***	-1.14***	-1.14***	-1.11***	-3.34**	-3.63**	-3.58**	-2.34*	-2.56*	-2.59**
	(0.38)	(0.38)	(0.37)	(0.40)	(0.40)	(0.39)	(1.54)	(1.58)	(1.44)	(1.30)	(1.32)	(1.24)
Number of countries (trial)	0.01	0.01	0.01	0.01	0.01	0.01	0.01	0.02	0.02	0.01	0.02	0.01
	(0.01)	(0.01)	(0.01)	(0.01)	(0.01)	(0.01)	(0.02)	(0.03)	(0.02)	(0.02)	(0.02)	(0.02)
Domestic sponsor	1.12**	1.15**	1.21**	1.52***	1.56***	1.61***	4.95	4.78*	4.63*	3.98**	3.86**	3.71**
	(0.50)	(0.50)	(0.50)	(0.45)	(0.45)	(0.47)	(3.06)	(2.77)	(2.37)	(1.97)	(1.80)	(1.54)
Trial start years	Yes	Yes	Yes	Yes	Yes	Yes	Yes	Yes	Yes	Yes	Yes	Yes
Publication years	Yes	Yes	Yes	Yes	Yes	Yes	Yes	Yes	Yes	Yes	Yes	Yes
Constant	-4.19***	-4.06***	-4.21***	-4.61***	-4.43***	-4.56***	-11.23***	-9.43***	-10.02***	-8.41***	-7.05***	-7.35***
	(1.46)	(1.47)	(1.47)	(1.44)	(1.46)	(1.47)	(3.82)	(3.26)	(3.03)	(2.54)	(2.22)	(2.02)
Sigma							3.55***	3.48***	3.33***	2.76***	2.69***	2.56***
							(0.45)	(0.41)	(0.35)	(0.36)	(0.31)	(0.25)
N	385	385	385	385	385	385	385	385	385	385	385	385
AIC	1019.31	1020.87	1021.04	950.13	951.39	951.74	1074.18	1069.88	1056.94	954.86	950.53	937.09
BIC	1118.14	1123.65	1127.77	1048.96	1054.18	1058.47	1176.96	1176.62	1167.63	1057.64	1057.26	1047.78

Robust standard errors in parentheses

* p<0.10,

** p<0.05,

*** p<0.01

As in the case of unweighted forward citations, our results for weighted forward citations suggest that Indian researchers are more likely to refer to phase 3 clinical trial-related publications that have been authored by Indian researchers than to those without Indian authors. When comparing trials with and without preceding phase 2 trials, we find significant differences in terms of the number of citations when we weight the number of subsequent citations by the number of countries involved (Models 8 and 11). However, the coefficient for *No Preceding Phase 2* loses significance when we introduce the interaction between *Indian Author* and *No Preceding Phase 2* to the analysis. Negative coefficients in Models 9 and 12 indicate that publications associated with phase 3 clinical trials without preceding phase 2 trials receive fewer citations by Indian authors. The results for the weighted citations counts remain qualitatively similar if ordinary least squares regressions are used to analyze weighted citation counts. Notably, our results are not driven by clinical trials of generic drugs or biosimilars as demonstrated by the auxiliary analysis described in the Methods section.

With respect to the control variables, we find a robust positive association between the presence of co-authors working for organizations from traditional clinical research countries (i.e., countries in North America, Western Europe, and selected Asia-Pacific locations) and citations by Indian authors.

Moreover, we find that phase 3 trials sponsored by biotechnology or pharmaceutical companies are cited less by Indian authors than trials sponsored by academic, healthcare, or philanthropic organizations. We also find that Indian authors more frequently cite trial-related publications that refer to trials sponsored by domestic companies.

## Discussion

This paper’s objective is to improve our understanding of how policy changes related to clinical trials affect the scientific knowledge base. An understanding of the complex dynamics in this context is important for effective regulation and, in our case, for understanding the consequences of relaxing clinical trial regulations. India serves as the context of our study, as the country has been a first-choice location for the offshoring of clinical trials and as it changed its clinical trial regulations in order to attract more late-stage (offshored) clinical trials.

The results of our empirical analyses suggest that the regulatory change (i.e., allowing phase 3 trials to be carried out even if they were not preceded by a phase 2 trial in the country) was followed by an initial increase in the number of phase 3 trials without preceding phase 2 trials, especially trials sponsored by Western pharmaceutical companies. However, the benefits for India’s domestic scientific knowledge base have been limited, as the involvement of India-based researchers in trial-related publication activities remained modest after the repeal of the phase lag requirement. In addition, the potential for learning from the additional trials appears to be limited, as publications referring to phase 3 trials without preceding phase 2 trials are less frequently used in Indian researchers’ subsequent research activities than publications related to phase 3 trials with preceding phase 2 trials. Overall, our findings contradict the expectation that the changes to the Indian clinical trial regulations enacted in January 2005 would effectively support knowledge transfer, the advancement of the domestic scientific knowledge base, and the development of new, innovative drugs by domestic companies. The increasing number of phase 3 trials without preceding phase 2 trials can be seen as a reflection of India’s growing attractiveness as a host country for clinical trials after the repeal of the phase lag requirement. India has been able to attract phase 3 trials sponsored primarily by foreign (i.e., predominantly Western) companies, especially in the years immediately after the repeal of the phase lag. The decreasing number of phase 3 clinical trials without preceding phase 2 trials in more recent years may be caused by dissatisfaction among trial sponsors and contract research organizations with India’s administrative and institutional environment as well as the perceived loss of credibility due to cases of fraud and poorly conducted trials [35].

The regulatory amendments provided only limited benefits to India’s science system. More specifically, the involvement of Indian researchers in trial-related scientific publications remains quite modest and knowledge generated by phase 3 trials without preceding phase 2 trials has a limited impact among domestic researchers compared to phase 3 trials with preceding phase 2 trials. In this regard, our results reveal that the benefits of offshored late-stage clinical trials for the domestic scientific knowledge base seem to be limited, as Indian researchers are still only involved in trial-related publication activities to a minor extent. One potential explanation for this finding is that Indian researchers’ access to clinical trial data and, hence, the opportunities they have to contribute to data analysis and publication activities may be considerably restricted for industry-sponsored trials, which represent the vast majority of phase 3 trials without a preceding phase 2 trials [36]. An additional explanation for the limited involvement of Indian researchers in trial-related publications is the, until recently, rather limited emphasis many Indian medical institutions put on publication activities. A recent study analyzing the publications of Indian medical institutions between 2005–2014 using Scopus, revealed that nearly 60% of Indian medical institutions had no single peer-reviewed publication in a decade [37]. While policy makers aim to increase publication rates, e.g., by increasing the minimum number of research publications that is required for promotion to the associate or full professor level [38], it takes time to establish an academic culture that emphasizes the importance of publications in (international) journals. In addition, Indian researchers may need to be acquainted with knowledge about habits and informal rules which spur successful publishing in international journals.

Our finding corresponds to earlier contributions stressing the limited involvement of researchers from non-traditional clinical trial countries in trial-related publication activities as well as the low number of publications by industry scientists working in R&D labs in non-traditional countries when compared to publications by their counterparts in Europe and North America [33,39]. Moreover, Phase 3 trials sponsored by biotechnology or pharmaceutical companies are associated with fewer citations by Indian authors than trials sponsored by academic, healthcare, or philanthropic organizations. This finding may be driven by concerns related to the influence of industry sponsors on the outcomes of clinical trials, the restricted dissemination of results in scientific publications, and scientific and ethical misbehavior among Indian companies conducting clinical trials [35,40].

In addition, we find that the number of forward citations made by Indian authors is lower for publications related to phase 3 trials that are not associated with a preceding phase 2 trials when all trials in our sample are considered. This indicates that the knowledge produced in offshored, late-stage clinical trials is only used by domestic researchers to a limited extent, as also expressed in the rather low number of forward citations of trial-related publications and the particularly low number of forward citations of publications associated with industry-sponsored trials.

However, the presence of co-authors working for organizations from traditional clinical research countries increases citations by Indian authors. This positive relationship might be driven by several factors. For example, researchers from traditional countries may have more experience in analyzing clinical trial data and in writing contributions for scientific journals that communicate trial results to the international scientific community. This experience may increase the quality of trial-related articles and, hence, the number of forward citations, including citations by Indian authors. In addition, researchers from traditional countries may self-select into clinical research projects that have a higher probability of success, a higher degree of novelty, and higher scientific quality. Moreover, these authors may share their publications within their professional networks which may increase the attention paid to those publications within the scientific community and, hence, increase the number of citations, including the number of citations by Indian authors.

Clinical trials sponsored by Indian organizations are likely to focus on disease areas that predominantly address health problems prevalent in India, which increases their relevance for domestic researchers and authors and, consequently, the number of citations by Indian researchers.

Taken together, our findings suggest that abolishing the phase lag reduces the ability of Indian researchers to learn from trials if the outsourced trial only focuses on Phase 3 and does not include Phase 2. A potential explanation for this seemingly limited knowledge transfer can be found in the nature of clinical trials. While clinical trials through phase 2 can be described as highly knowledge-intensive [41] trials that may offer opportunities for knowledge transfer to Indian researchers, late-stage trials focused on data generation provide fewer opportunities for knowledge transfer. Consequently, it seems much more difficult for Indian researchers to learn from offshored phase 3 trials that are not accompanied by preceding phase 2 trials. As a consequence, Indian researchers cite publications that refer to phase 3 trials that are not associated with preceding phase 2 trials less frequently than trials with preceding phase 2 trials. In addition, the limited involvement of Indian researchers in publication activities related to late-stage clinical trials may further increase the complexity of knowledge transfers and complicate the application of trial-related knowledge in their own clinical research projects. The consequences of this limited involvement may reach beyond the individual researcher, as they also may make the dissemination of knowledge embodied in trial-related publications through Indian researchers’ professional networks less likely and less effective.

Based on these results and interpretations, the Indian case does not suggest that lower regulatory hurdles for late-stage clinical trials provide the expected benefits. Consequently, the Indian case may not be the best role model for other non-traditional countries wishing to support the development of their domestic scientific knowledge bases through clinical research. Instead, our results suggest that governments need to search for alternative ways of promoting knowledge transfers and the development of the domestic scientific knowledge base, such as investments in domestic scientific and technological capabilities to strengthen the country’s absorptive capacity or to attract multinational companies’ R&D labs.

## Limitations

It is important to emphasize that our analyses focus on the consequences of the repeal of the phase lag requirement for India’s domestic scientific knowledge base in terms of scientific publications and forward citations. Future research may address the impact of this regulatory change on India’s abilities to attract talent and to encourage Indian scientists to return from abroad, as well as its impact on the establishment of knowledge-intensive R&D service companies, FDI, and the direction of domestic research activities. Along similar lines, future research may study the consequences of regulatory changes on other dimensions of the domestic scientific knowledge base such as the number of domestic researchers in disciplines that are relevant for clinical research, their training and education, their involvement in clinical trials, or changes in domestic R&D investments that have not been the focus of this study. Another area of research might be the consequences of this repeal for the availability of new pharmaceuticals to the domestic population.

Our empirical analyses build on data covering clinical trials and trial-related publications that are listed in international databases. This data may not fully account for trial-related publications by Indian authors in domestic journals. Future research may assess whether the consequences of changing clinical trial regulations differ when domestic databases are considered. Moreover, our data do not allow us to assess the role Indian researchers play in trial-related publications (i.e., whether they are involved in different scholarly activities and whether they solely contribute data) or the extent to which clinical trial sponsors restrict domestic researcher from accessing data and publishing trial results. Future research may also assess the consequences of outsourcing trial-management tasks to contract research organizations for the domestic scientific knowledge base.

ClinicalTrials.gov provides a comprehensive registry for clinical trials. However, there might be trials that have been affected by India’s regulatory change but have not been registered in ClinicalTrials.gov. Instead these trials may have been registered in other registries as the compounds tested in these trials did not meet the requirements for mandatory registration in ClinicalTrials.gov or the trial sponsor did not intend to market the compound in the United States once clinical testing had been completed. Since India’s domestic registry for clinical trials had not been established yet around the time of the regulatory change studied in this paper, we could not supplement the data obtained from ClinicalTrials.gov with data on trials that were only registered domestically.

ClinicalTrials.gov employs automated and manual reviews to identify possible errors, deficiencies, or inconsistencies in the information provided by trial sponsors or investigators. There are considerably penalties for noncompliance with the rules of clinical trial registration [42]. Nevertheless, not all trials are subject to manual reviews and checks and our study has to rely on the accuracy of the information that trial sponsors and investigators report to the database. While the ClinicalTrials.gov provides a list of facilities that the trial is conducted in, it does not contain information concerning the recruitment of subjects on the facility level. Hence, it is not possible to verify whether trial subjects have been recruited in a particular facility or how important a specific location was for recruiting subjects.

Given the above discussion, our analysis serves as a starting point for a debate on how non-traditional countries’ science systems can benefit from the internationalization of clinical trials. As part of this debate, researchers need to carefully evaluate whether offshored clinical research provides other benefits for non-traditional countries’ science and health-care systems.

## Supporting information

S1 Table Correlations among independent variables. (DOCX)

S2 Table Data on phase 3 clinical trials conducted in India. (TXT)
