# Supplementary material for: Did relaxing clinical trial regulation enhance the stock of scientific knowledge in India? Not necessarily
Source: PLoS One. 2019 Jan 3;14(1):e0210163. doi: 10.1371/journal.pone.0210163 (PMC6317798; doi:10.1371/journal.pone.0210163)
Supplement: S1 Table — (DOCX) [file pone.0210163.s001.docx]

|  |  | (1) | (2) | (3) | (4) |
| --- | --- | --- | --- | --- | --- |
| (1) | Indian Author | 1.00 |  |  |  |
| (2) | No Preceding Phase 2 | -0.01 | 1.00 |  |  |
| (3) | Basic Research Journal | 0.08 | 0.09 | 1.00 |  |
| (4) | Number of Authors | 0.07 | 0.01 | 0.07 | 1.00 |
| (5) | Page Count | 0.00 | 0.01 | -0.05 | 0.10* |
| (6) | Number of Countries (Publication) | 0.18*** | -0.05 | -0.13* | 0.32*** |
| (7) | Traditional Country Co-author | -0.31*** | 0.07 | 0.03 | 0.05 |
| (8) | Life Threatening Disease | -0.05 | -0.12* | -0.03 | 0.00 |
| (9) | Industry Sponsor | -0.56*** | -0.05 | -0.01 | -0.04 |
| (10) | Number of Countries (Trial) | -0.38*** | 0.10 | -0.03 | 0.08 |
| (11) | Domestic Sponsor | 0.49*** | 0.02 | 0.04 | -0.04 |
|  |  |  |  |  |  |
|  |  | (5) | (6) | (7) | (8) |
| (5) | Page Count | 1.00 |  |  |  |
| (6) | Number of Countries (Publication) | 0.11* | 1.00 |  |  |
| (7) | Traditional Country Co-author | 0.13* | 0.20*** | 1.00 |  |
| (8) | Life Threatening Disease | 0.02 | 0.09 | 0.03 | 1.00 |
| (9) | Industry Sponsor | 0.11* | 0.04 | 0.43*** | 0.12* |
| (10) | Number of Countries (Trial) | 0.06 | 0.37*** | 0.26*** | 0.11* |
| (11) | Domestic Sponsor | -0.17** | -0.19*** | -0.67*** | -0.13** |
|  |  |  |  |  |  |
|  |  | (9) | (10) | (11) |  |
| (9) | Industry Sponsor | 1.00 |  |  |  |
| (10) | Number of Countries (Trial) | 0.43*** | 1.00 |  |  |
| (11) | Domestic Sponsor | -0.64*** | -0.41*** | 1.00 |  |
|  | * p<0.05, ** p<0.01, *** p<0.001 |  |  |  |  |
